# Supplementary material for: Epigenetic changes associated with the progression of prion disease in Syrian hamsters (Mesocricetus auratus)
Source: Prion. 2026 Aug 10;20(1):52–65. doi: 10.1080/19336896.2026.2710965 (PMC13471106; doi:10.1080/19336896.2026.2710965)
Supplement: Supplementary file 1 [file KPRN_A_2710965_SM9011.docx]

**Supplementary Data - Epigenetic Changes Associated with the Progression of Prion Disease in Syrian Hamsters (Mesocricetus auratus)**

Lexi E. Frank1,2*, Nicole Flack1,2, Christopher Faulk3, Alyssa J. Block4, 5, Jason C. Bartz2,4, Peter A. Larsen1,2

1 Department of Veterinary and Biomedical Sciences, University of Minnesota, St. Paul, MN, U.S. 55108, 2 Minnesota Center for Prion Research and Outreach, St. Paul, MN, U.S. 55108, 3 Department of Animal Science, University of Minnesota, St. Paul, MN, U.S. 55108, 4 Department of Medical Microbiology and Immunology, School of Medicine, Creighton University, Omaha, NE, U.S., 5 Current Address: Department of Microbiology, Immunology, and Pathology, Colorado State University, Fort Collins, CO, U.S. 80523

*Corresponding Author

Lexi E. Frank

lefrank1464@gmail.com

Department of Veterinary and Biomedical Sciences

1971 Commonwealth Ave. St. Paul, MN 55108 OCRiD: 0009-0006-7736-4146

*Prion*

**Supplementary Data 1. Detailed Bioinformatic Processing Workflow.**

supplementarydata1.html

**Supplementary Data 2. Summary of sequencing and alignment statistics for treatment and control samples.** Mean genome coverage, N50, and Mean identity of sequence alignment to the reference genome are shown for each sample. The treatment (T = treatment, C = control) and time point ( 1 = 80 dpi, 2 = 120 dpi, 3 = 160 dpi) are indicated in the Treatment and Time Point column.

| **Sample #** | **Treatment and Time Point** | **Mean Genome Coverage** | **N50 (bases)** | **Median identity (%)** |
| --- | --- | --- | --- | --- |
| 1 | T1 | 23.5 | 3822 | 98.57 |
| 2 | T3 | 21.89 | 4437 | 98.58 |
| 3 | C1 | 23.55 | 5049 | 98.42 |
| 4 | T1 | 20.25 | 5162 | 98.4 |
| 5 | T1 | 19.51 | 4286 | 98.54 |
| 6 | C3 | 19.55 | 4492 | 98.44 |
| 7 | T3 | 19.98 | 5553 | 98.23 |
| 8 | C2 | 21.84 | 4865 | 98.6 |
| 9 | C2 | 22.5 | 4412 | 98.68 |
| 10 | T2 | 21.4 | 4398 | 98.5 |
| 11 | C3 | 21.1 | 4395 | 98.54 |
| 12 | T2 | 19.74 | 3350 | 98.2 |
| 13 | C1 | 19.44 | 4398 | 98.51 |
| 14 | T2 | 20.3 | 4256 | 98.39 |
| 15 | C1 | 21.3 | 3612 | 98.36 |
| 16 | C2 | 21.84 | 3862 | 98.7 |
| 17 | C3 | 20.66 | 3905 | 98.64 |
| 18 | T3 | 21.2 | 2983 | 98.52 |

**Supplementary Data 3.** Global methylation levels of *Mesocricetus auratus* brain tissues. Percent of CpGs being canonical cytosine, 5-methyl-cytosine, and 5-hydroxy-methyl-cytosine. T = treatment group, C = control group. 1 = time point 1 at 80 dpi, 2 = time point 2 at 120 dpi, and 3= time point 3 at 160 dpi.

| **Sample #** | **Treatment and Time Point** | **CpG Canonical (%)** | **5mCpG Modified (%)** | **5hmCpG Modified (%)** |
| --- | --- | --- | --- | --- |
| 1 | T1 | 29.19 | 60.59 | 10.23 |
| 2 | T3 | 29.36 | 60.32 | 10.32 |
| 3 | C1 | 28.77 | 60.73 | 10.49 |
| 4 | T1 | 29.54 | 60.28 | 10.18 |
| 5 | T1 | 29.86 | 60.34 | 9.80 |
| 6 | C3 | 29.03 | 60.73 | 10.24 |
| 7 | T3 | 29.80 | 60.43 | 9.77 |
| 8 | C2 | 29.66 | 60.46 | 9.88 |
| 9 | C2 | 30.08 | 59.87 | 10.05 |
| 10 | T2 | 30.68 | 60.18 | 9.13 |
| 11 | C3 | 30.35 | 59.51 | 10.14 |
| 12 | T2 | 30.31 | 60.11 | 9.58 |
| 13 | C1 | 30.42 | 59.95 | 9.63 |
| 14 | T2 | 30.87 | 59.13 | 10.00 |
| 15 | C1 | 30.92 | 59.52 | 9.55 |
| 16 | C2 | 30.02 | 59.59 | 10.39 |
| 17 | C3 | 29.78 | 59.92 | 10.30 |
| 18 | T3 | 31.62 | 59.14 | 9.24 |

**Supplementary Data 4. Genomic distribution of differentially methylated regions (DMRs) across promoter, exon, and intron features at 80, 120, and 160 days post-inoculation (dpi).** Shown are the proportions of DMRs relative to the genomic background and associated chi-square test p-values, indicating significant enrichment or depletion of DMRs within specific genomic features over the course of disease progression.

| **Timepoint** |  | **Promoter** | **Exon** | **Intron** |
| --- | --- | --- | --- | --- |
| 80 dpi | DMRs | 3.59 | 10.97 | 40.76 |
|  | Background | 7.16 | 10.31 | 30.07 |
|  | chi square | 8.93E-08* | 0.4191 | 2.20E-16* |
| 120 dpi | DMRs | 4.14 | 12.36 | 39.1 |
|  | Background | 7.16 | 10.31 | 30.07 |
|  | chi square | 2.90E-06* | 7.39E-03* | 2.82E-15* |
| 160 dpi | DMRs | 4.26 | 13.37 | 42.08 |
|  | Background | 7.16 | 10.31 | 30.07 |
|  | chi square | 7.36E-08* | 1.59E-06* | 2.20E-16* |

**_* - indicates significance of p<0.05._**

**Supplementary Data 5. Gene Ontology (GO) enrichment analysis of genes nearest differentially methylated regions and differentially expressed genes.** Enriched biological process (BP) terms calculated with DAVID are shown with corresponding GO identifiers, number of genes mapped to each term (Count), percentage of annotated genes, enrichment *p*-values, and multiple testing corrections (Bonferroni, Benjamini, and FDR). “Genes” lists identifiers of the differentially methylated genes contributing to each enriched term. “List Total” refers to the total number of differentially methylated genes tested, while “Pop Hits” and “Pop Total” indicate the number of annotated genes and the total population background, respectively. Fold enrichment values represent the ratio of observed to expected counts under a hypergeometric distribution.

supplementarydata5.csv

**Supplementary Data 6. Venn diagrams showing overlap of differentially expressed genes across time points.** Upregulated (left) and downregulated (right) genes were identified at 80, 120, and 160 days post-inoculation (dpi). The majority of differentially expressed genes were unique to a single time point, with limited overlap observed between stages of disease progression. Notably, the largest number of both upregulated and downregulated genes occurred at 160 dpi, indicating that transcriptional changes intensify during late-stage disease.
